# Estimating the Fitness Effects of New Mutations in the Wild Yeast *Saccharomyces paradoxus*

**DOI:** 10.1093/gbe/evv112

**Published:** 2015-06-16

**Authors:** Vassiliki Koufopanou, Susan Lomas, Isheng J. Tsai, Austin Burt

**Affiliations:** ^1^Department of Life Sciences, Imperial College London, Silwood Park, Ascot, Berks, United Kingdom; ^2^Present address: Biodiversity Research Center, Academia Sinica, Taipei, Taiwan

**Keywords:** purifying selection, derived allele frequency, deleterious mutations, fitness effects of mutations

## Abstract

The nature of selection acting on a population is in large measure determined by the distribution of fitness effects of new mutations. In this study, we use DNA sequences from four closely related clades of *Saccharomyces paradoxus* and *Saccharomyces cerevisiae* to identify and polarize new mutations and estimate their fitness effects. By progressively restricting the analyses to narrower categories of sites, we further seek to characterize sites with predictable mutational effects, that is, unconditionally deleterious, neutral or beneficial. Consistent with previous studies on *S. paradoxus*, we have failed to find evidence for mutations with beneficial effects, even in regions that were divergent in two outgroup clades, perhaps a consequence of the relatively unchallenged, predominantly asexual and highly inbred lifestyle of this species. On the other hand, there is abundant evidence of deleterious mutations, varying in severity of effect from strongly deleterious to very mild, particularly in regions conserved in the outgroup taxa, indicating a history of persistent purifying selection. Narrowing the analysis down to individual amino acids reduces further the range of effects: for example, mutations changing cysteine are predicted to be nearly always strongly deleterious, whereas those changing arginine, serine, and tyrosine are expected to be nearly neutral. The proportion of mutations with deleterious effects for a particular amino acid is correlated with long-term stasis of that amino acid among highly divergent sequences from a variety of organisms, showing that functionality of sites tends to persist through the diversification of clades and that our findings are also relevant to longer evolutionary times and other taxa.

## Introduction

The distribution of fitness effects of new mutations plays a central role in evolutionary biology, as it provides insights into the genetic architecture of traits and also on their past selective history. The likelihood of new mutations being advantageous, neutral or deleterious will depend on the previous selective history of the affected traits, advantageous mutations being rare where natural selection has been acting consistently in the past, fixing most of the available beneficial variants and more frequent where selection was more variable. Ultimately, mapping where the advantageous, neutral, and deleterious mutations are likely to occur provides critical information on functionality and disease, and is therefore of both theoretical and practical interests.

Traditionally, phenotypic effects of mutations have been measured in the laboratory, in mutation accumulation experiments where populations were maintained under relaxed selection for several generations, or following a mutagenesis treatment (e.g., Mukai et al. 1972; Keightley and Ohnishi 1998; Keightley et al. 2000; Vassilieva et al. 2000; Wloch et al. 2001; studies reviewed in Eyre-Walker and Keightley 2007). Such experiments have revealed a predominantly deleterious range of phenotypic effects for new mutations in several traits and only a very low frequency of advantageous effects, suggesting a history of conservative adaptation in populations, maintained by purifying selection, with rare progressive steps. Life-history traits seem the most adversely affected by mutation, indicative of a long history of directional selection (Keightley and Ohnishi 1998). More recently, fitness effects of experimentally induced mutations in particular regions of proteins have been estimated in vitro in yeast (Eyre-Walker and Keightley 2007; Bank et al. 2014; Melamed et al. 2014). Finally, experiments have also shown an extensive genotype-by-environment interaction, indicating that the effects of mutations are also modulated by the environment (Bell 2008), and that conducting fitness assays in the novelty of the laboratory environment may fail to capture at least some of the effects in nature.

The recent extensive availability of DNA sequences has allowed estimation of genomic rates of mutation from direct measurements of nucleotide diversity among individuals in situ, in the organisms natural environments, and insights into the nature of adaptation through comparisons of rates of polymorphism and divergence between regions presumed to be under different selective regimes (studies reviewed in Eyre-Walker 2006), but the relative contributions of a positive, Darwinian process whereby an organism adapts to an ever-changing environment, as opposed to more conservative evolution, primarily maintaining current adaptation by removal of deleterious mutations, remains elusive. The wild yeast *Saccharomyces paradoxus* is well suited for studies in population genomics, showing extensive population differentiation among several lineages (contrasting with a fairly homogeneous global distribution in the domesticated *Saccharomyces cerevisiae*; Koufopanou et al. 2006; Replansky et al. 2008; Liti et al. 2009). Within lineages, populations are well mixed, though sexual generations appear to be infrequent (Johnson et al. 2004; Tsai et al. 2008). Previous studies have shown little evidence of positive Darwinian selection in *S. paradoxus* (Elyashiv et al. 2010; Vishnoi et al. 2011; Gossmann et al. 2012), but abundant evidence of purifying selection, implying that a large fraction of the genome is functional.

**Fig. 1.—:**
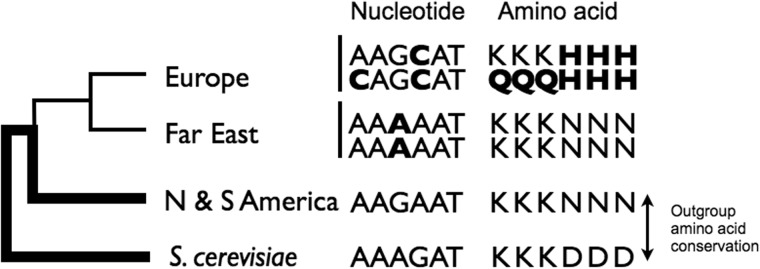
Phylogenetic relationships among the three lineages of *S. paradoxus* and *S. cerevisiae* illustrating the assignment of DA status (DAs shown in bold) and outgroup amino acid conservation.

This study uses four independent clades of *S. paradoxus* and relatives to identify new mutations in one population and estimate their fitness effects. We use one clade to measure polymorphism, and two outgroups to establish the derived state of alleles, the closest outgroup to polarize the polymorphisms, and the next for the fixed alleles. Finally, a fourth clade (outgroup to the others) is used to characterize the conservation status of sites (fig. 1). We are applying a method developed by Schneider et al. (2011), which compares the frequency distributions of newly derived alleles (DAs), between genomic regions presumed to be undergoing selection and others that can be assumed to be relatively neutral. An excess or deficiency of new mutations in “selected” compared with “unselected” regions is used to estimate the extent to which mutations might be advantageous or deleterious. By progressively restricting the analysis to narrower categories of sites, we further seek to identify sites where the mutational effects are predictably deleterious, neutral, or beneficial.

## Materials and Methods

### Strains Analyzed

A global alignment of 300,538 nt was analyzed for chromosome III, including 12 strains from the European population, 8 from Far East, and 1 of *Saccharomyces cariocanus*, aligned to the *S. cerevisiae* reference sequence, as described previously (Bensasson et al. 2008; Tsai et al. 2008). The European population includes strains from Berkshire, United Kingdom, collected within a 10-km^2^ area, plus the published sequence for the European Type strain of *S. paradoxus* (CBS 432; Kellis et al. 2003). There is no evidence of geographic differentiation between these 12 strains (Johnson et al. 2004), though there is some evidence of differentiation of strains from a wider sample within Europe (Koufopanou et al. 2006).

### Annotation

Only the “verified” and “uncharacterized” categories of genes in the Saccharomyces Genome Database were used to annotate our *S. paradoxus* chromosome III alignment, that is, “dubious” open reading frames are not used. The alignment starts with the first 5′ base of YCL069W (VBA3) and ends with the first 5′ base of YCR095C (OCA4). A total of 135 genes were analyzed, comprising a total of 194,618 nt (out of a total of 143 coding annotations in *S. cerevisiae*; 3 uncharacterized gene annotations, YCL001W-A, YCR024C-B, YCR089W, were not used due to the existence of multiple premature stop codons in the *S. paradoxus* sequences in these genes); annotation for the YCL042W *S. cerevisiae* locus was not used because it overlaps with the verified annotation of YCL040W.

In cases of changes in the start or end positions between *S. paradoxus* and *S. cerevisiae*, the alignment was adjusted to preserve the start/end codons in *S. paradoxus* (so that no premature stop codons exist in *S. paradoxus*). A total of 11 genes had different start/end positions (due to small indels, premature codons or extensions, all within 50 bp from either end: YCL068C, YCL049C, YCL001W-B, YCR015C, YCR018C, YCR044C, YCR073W-A, YCR092C; multiple consecutive start codons in *S. cerevisiae* and only one in *S. paradoxus*: YCR038C, YCR042C, YCR073C). Finally, in the coding region of YCR028C there is polymorphism among European strains for a premature stop codon, resulting in loss of 5 of 512 amino acids. Long Terminal Repeat (LTR) annotations are as described previously (Tsai et al. 2008).

### Divergence and Polymorphism

The ancestral state of polymorphic alleles in the European population was inferred using the Far East population as outgroup; when different alleles were fixed in the two populations, *S. cariocanus* was used as outgroup. The number of DAs was calculated using Mathematica, and coding of 0-, 2-, and 4-fold degenerate sites of coding regions was done using the MEGA software (Tamura et al. 2013).

## Results and Discussion

### Purifying Selection on Coding Sites

To measure polymorphism, we used an alignment of DNA sequences from the third chromosome of individuals in the European clade of *S. paradoxus*. To infer the ancestral status of alleles, we compared the European sequences with those from the Far Eastern population of the same species; for fixed differences, or when the Far East homologue was missing, we used the North American clade. Note that for the overwhelming majority of sites where DAs were inferred, both the Far East and North American lineages indicate the same allele as ancestral, thus providing further confidence in estimating the direction of change (e.g., 97% of coding sites with DAs). Previous analyses have shown mixing and lack of geographic differentiation within our sample of European strains (all but one from the United Kingdom; see also Materials and Methods), a critical requirement for estimating fitness effects. There is 1.4% overall nucleotide divergence between the European and Far East populations, with LTRs of transposable elements being the fastest evolving regions, consistent with their nonfunctional status (Bensasson et al. 2008), and coding replacement sites the slowest (4.6% and 0.5% divergence, respectively), indicating that the overall net effect of selection is to slow down evolution. Rates of polymorphism are about ten times lower than divergence.

**Fig. 2.—:**
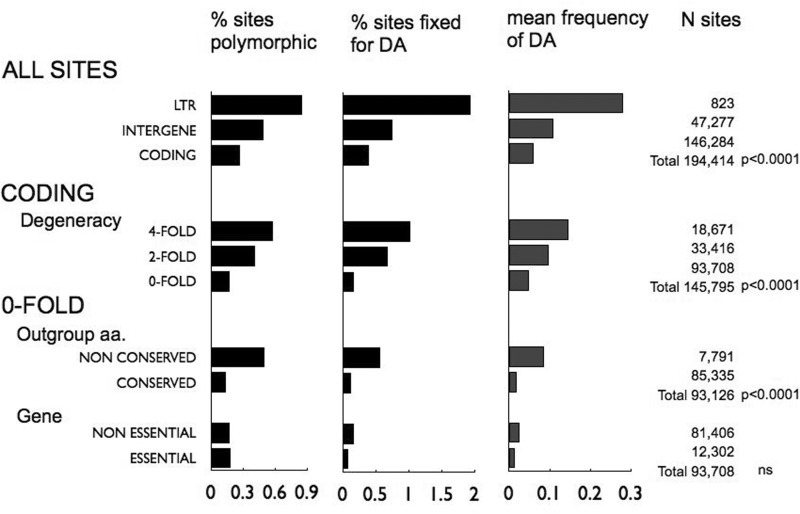
Frequency distributions of DAs in the European population, at different categories of sites (probabilities are from nonparametric Wilcoxon tests for differences among category means). Derived status of alleles in Europe was assigned by comparison to the Far East population; for sites fixed in Europe, by comparison to the *S. cariocanus* allele. Note only sites with no missing values in the European population are included in the analysis, and for which the DA status could unambiguously be determined (i.e., there were data present for at least one strain from the Far East population and there were data for the *S. cariocanus* strain, and no ambiguity in status assignment). To remove any effects of spatial autocorrelation within the chromosome due to some consecutive sites along the chromosome being compared with other consecutive sites, the comparison between essential and nonessential genes was performed on the means of individual genes (*n* = 12 and 113, respectively); all other comparisons have sites scattered among categories. LTR: Long Terminal Repeat regions, remnants of transposable elements presumed to be nonfunctional; only LTRs fixed between Europe and Far East are analyzed here, to exclude recent inserts that would not be comparable with the rest of the chromosome.

Of 194,384 sites included in the analysis, 13,776 sites could unambiguously be characterized as having DAs in the European population, including sites fixed for the DA, giving an overall frequency of 0.07 per site in the alignment (and including those fixed for the ancestral allele, i.e., invariant sites with 0 DAs; note only sites with no missing data were analyzed). There is significant difference in the mean frequency of DAs between different types of genomic regions, with LTRs having the highest, and coding regions the lowest frequencies (mean frequency of DAs per site: 0.28 vs. 0.11 vs. 0.06, for LTR, intergene and coding, respectively; Wilcoxon nonparametric test, *P* < 0.0001; fig. 2). Within coding regions, 4-fold degenerate sites (at which changes in the nucleotide do not affect the amino acid encoded) had lower average frequency of DAs than LTRs, indicating some purifying selection at these synonymous sites, as we have found previously (Bensasson et al. 2008). Evidence suggesting selection at synonymous sites has been shown in *Drosophila*, humans, and bacteria (Eory et al. 2010; Lawrie et al. 2013; Bailey et al. 2014). Nondegenerate sites were lower still, having only about one-third the average DA frequency as the 4-fold degenerate sites (0.15 vs. 0.10 vs. 0.05, for 4-, 2-, and 0-fold degenerate sites, respectively, *P* < 0.0001; fig. 2). This was reflected in a lower frequency of both segregating polymorphisms and fixed differences. Similar results were also found in a previous study of *S. paradoxus*, where frequencies of DAs were compared between categories of sites differing in functionality (Vishnoi et al. 2011). In contrast to our study, however, where only one lineage is in focus and the rest used as outgroups to polarize changes, Vishnoi et al. apparently ignored the subdivision and pooled all reproductively isolated lineages together.

### Estimating the Distribution of Fitness Effects of Newly Derived Mutations

#### Description and Applicability of the Model

The program of Schneider et al. (2011) takes as input the distributions of DAs in two categories of sites, an “unselected” category (i.e., sites assumed to be under no selection or nearly so), and a “selected” category, and from these calculates the likelihoods of the data under different scenarios for magnitude and distribution of fitness effects (Distribution of Fitness Effects, DFE-Adaptive Server; http://www.homepages.ed.ac.uk/pkeightl/, last accessed May 2015; Schneider et al. 2011).

To select a model that adequately describes the population, the program examines first whether there is any evidence for change in population size during the divergence of the two populations from their common ancestor, by comparing the fit of models with and without constant population size, to the distribution of DAs in the unselected category of sites. We have used here the 4-fold degenerate sites as the unselected category, even though there is evidence for some purifying selection, above, as there are not sufficient data for the LTRs. Using these data, there is no evidence for a change in population size, so we assumed constant size for the subsequent analyses (2log L(change) − 2log L(constant) = 0.33, *P* > 0.5, for a model allowing change in size of the European population during its divergence from the Far East population vs. one with constant population size).

The program then uses parameters estimated from the unselected sites distribution to estimate the proportion of advantageous mutations within the selected category of sites, and a mean effect of advantageous mutations. Note that it is the product of these two parameters that is estimated with the highest accuracy, even for small sample sizes (larger data sets are required to estimate each of the two parameters separately; Schneider et al. 2011). For deleterious mutations, the program assumes that the effects at different sites are either variable among sites, following a gamma distribution, and estimates the parameters for that distribution, (i.e., a mean effect and a shape parameter beta), or equal at all sites, and estimates the average effect. These parameters can be estimated reasonably accurately for sample sizes similar to those used here (<10% deviations in the estimated parameters for sample sizes as small as 2,500 sites; Schneider et al. 2011). The program also estimates the proportions of substitutions with different magnitudes of deleterious effect, from effectively neutral to strongly deleterious. The mean effects of both advantageous and deleterious mutations are estimated as products of the effective population size, that is, N_e_s_a_ or N_e_S_d_, for advantageous and deleterious effects, respectively. The estimates of the mean parameter of gamma, N_e_S_d_, should be interpreted with some caution as they tend to be noisy (e.g., table 5 in Keightley and Eyre-Walker 2007), and for this reason we have chosen to focus the analysis on estimated proportions of deleterious effects, which are more robust, rather than mean estimates (Keightley and Eyre-Walker 2007).

The Schneider et al. (2011) model assumes that all nucleotides are in linkage equilibrium, and we have shown previously that there is some linkage disequilibrium in our data, and perhaps subdivision introduced by the asexual generations and inbreeding (although we are analyzing a well-mixed population with no evidence of geographic differentiation; Tsai et al. 2008). Simulation studies, however, testing this and similar models have shown that estimates of the shape parameter beta of the gamma distribution are robust to deviations from free recombination, as long as linkage between sites is not complete, and to moderate population subdivision (table 5 in Eyre-Walker and Keightley 2009). Linkage disequilibrium between sites on chromosome III in the European population decays to 0 for sites that are more than 25 kb apart, and there is an average of 1 recombination event per kb along the entire chromosome (rho ranges between 3.1 and 1.1 Morgans/kb, depending on the method of estimation; Tsai et al. 2008); the average length of fragments for which there is no evidence of recombination is approximately 2.2 kb (haplotype blocks—see fig. 1 in Tsai et al. [2010]).

#### Parameter Estimation

For the 0-fold degenerate sites, the best model shows no sites under positive selection (equal likelihood of models with advantageous mutations fixed at nearly zero, or allowed to vary: Models 2 and 4, vs. 7; table 1), but an abundance of sites undergoing purifying selection (greatly decreased likelihood of models with deleterious effects set to 0 compared with when they are allowed to vary: Model 5 vs. all other models; table 1). By comparison, Schneider et al. (2011) applied their method to data from two populations of *Drosophila* and estimated that 1–2% of new nonsynonymous mutations are positively selected. Deleterious effects in the yeast sequences are best described by a leptokurtic gamma distribution with an estimated 32% mutations strongly deleterious (N_e_S_d_ > 100) and about 20% effectively neutral (N_e_S_d_ < 1). In this analysis, the model with variable deleterious fitness effects fits the data much better than one with equal fitness effects (model 2 vs. 1, *P* < 0.001; table 1). This result raises the question of whether we can find predictors of the fitness effects of new mutations at different sites, addressed in the sections below.

**Table 1:** Log Likelihoods of Different Models for the Distribution of Fitness Effects, for all 0-Fold Sites, and Separately for Outgroup-Conserved and Nonconserved Categories of Sites

Model	Parameter Input		Parameters Estimated	*n*	Total	Conserved	Nonconserved
	Advantageous Effects	Deleterious Effects			(93,708 sites)	(85,327 sites)	(7,721 sites)
1	None (p_a_ fix 0)	All equal	N_e_S_d_	1	−2,666.78	−1,995.85	−590.47
2	None (p_a_ fix 0)	γ-distributed	(N_e_S_d_, β_d_)	2	−2,603.3	−1,926.67	−590.47
3	∼None (s_a_ fix∼0)	All equal	N_e_S_d_, p_a_	2	−2,606.95	−1,929.54	−590.47
4	∼None (s_a_ fix∼0)	γ-distributed	(N_e_S_d_, β_d_), p_a_	3	−2,603.3	−1,926.67	−590.47
5	Variable	None (N_e_S_d_ fix 0)	p_a_, s_a_	2	−3,329.98	−2,691.1	−600.29
6	Variable	All equal	N_e_S_d,_ p_a_, s_a_	3	−2,606.95	−1,929.54	−590.47
7	Variable	γ-distributed	(N_e_S_d,_ β_d_), p_a_, s_a_	4	−2,603.3	−1,926.67	−590.47

Comparisons							
	Test	Models		df	dLog L (*P*)	dLog L (*P*)	dLog L (*P*)

	Advantageous ≠ 0	2 versus 7		2	0 (NS)	0 (NS)	0 (NS)
	Advantageous ≠ 0	4 versus 7		1	0 (NS)	0 (NS)	0 (NS)
	Deleterious ≠ 0	5 versus 7		2	726.68 (<0.001)	764.43 (<0.001)	9.82 (<0.01)
	Deleterious variable	1 versus 2		1	63.48 (<0.001)	69.18 (<0.001)	0 (NS)

Note.—*n*, number of parameters estimated by the model; *P*_a_, proportion of advantageous mutations; s_a_, average effect of advantageous mutations; note that fixing s_a_ to exactly 0 reduces the fit of the equal-effects model for deleterious mutations significantly, whereas it has no effect on the γ-distributed model; N_e_S_d_, the product of effective population size N_e_, times the average effect of deleterious mutations S_d_; for the equal-effects model, the program estimates the average N_e_S_d_; for the γ-distributed model, the two parameters of the γ distribution are estimated, that is, mean N_e_S_d_ and the shape parameter β_d_.

### The Impact of Mutations at Sites of Increased Constraint

Sites may differ among each other in their degree of tolerance to new mutations (evolvability), certain sites being very highly constrained due to some unique functionality conferred at those sites by one or very few particular amino acids, and therefore only accepting those amino acids. Such sites are expected to be under strong purifying selection and should be identifiable by the highly conserved status of their homologs in ancestral clades (assuming the same functionality persists during the diversification of clades). We have therefore categorized sites according to whether or not the orthologous amino acid-translated codon is conserved between two outgroup sequences, *S. cariocanus* and *S. cerevisiae* (hereto referred to as outgroup conserved vs. nonconserved sites; fig. 1). We find outgroup conserved sites to have significantly lower frequencies of DAs than nonconserved sites (*P* < 0.0001; fig. 2). We also looked at the effect of gene functionality, comparing between sites at essential genes, that is, genes whose deletion is lethal (Deutschbauer et al. 2005), versus those in nonessential genes, but there was no significant difference (fig. 2).

**Fig. 3.—:**
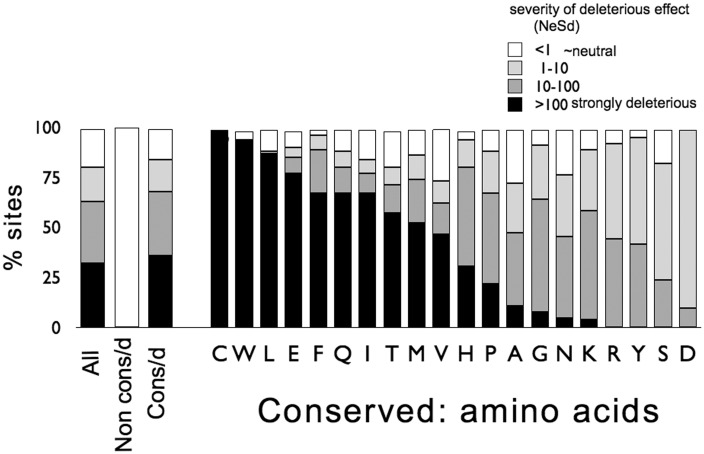
Proportions of sites at different categories of severity of deleterious effect caused by mutations at outgroup conserved and nonconserved sites, and for each amino acid separately, by mutations changing that amino acid; effects range from effectively neutral to strongly deleterious (note that deleterious effects are multiplied by the effective population size, i.e., N_e_S_d_ < 1 to N_e_S_d_ > 100). Only 0-fold degenerate sites are analyzed here; amino acids are ranked according to severity of deleterious effects (outgroup conserved sites only); note that the lethal or nearly lethal category of mutations is not included here, as such individuals will most likely be dead and thus not observable in the study.

For sites that differ between the two outgroup species, *S. cariocanus* and *S. cerevisiae*, the difference might be due to adaptive divergence in either one or both species, or to neutral drift. If the difference was due to adaptive divergence, and assuming similar patterns of selection across all clades, we would expect to see selection at the othologs of these sites in *S. paradoxus*, fixing advantageous or removing deleterious alleles. However, we found little evidence for selection of either type at those sites: No evidence for beneficial mutations (equal likelihoods for models with advantageous effects fixed at nearly zero and those with effects allowed to vary in frequency or magnitude: Models 2 and 4 vs. 7, *P* > 0.05; table 1), and no evidence for significantly deleterious mutations (in a model of purifying selection, the mean selection coefficient was small, N_e_S_d_ ∼ 0.53, with no evidence for significant variation among sites; table 2 and fig. 3). This lack of evidence for selection in the *S. paradoxus* orthologs of the outgroup-divergent sites suggests that most of the amino acid differences between *S. cariocanus* and *S. cerevisiae* may be due to drift rather than selection.

**Table 2:** Parameters Estimated from Models 1 and 2 (see table 1) for 0-Fold Degenerate Sites

	*N* Sites	Model 1	Model 2		ΔLog L	
		Deleterious Effects	Deleterious Effects		(Model 2 − Model 1)	
		All Equal	γ-Distributed		(df = 1)	
		N_e_S_d_	N_e_S_d_	β_d_		*P*
Total	93,708	1.64	140.3	0.28	63.48	<0.001
Outgroup Nonconserved	7,721	0.53	0.53	100	0	NS
Outgroup Conserved	85,327	1.87	158.68	0.33	69.18	<0.001
Ancestral AA^a^						
A	4,792	1.34	36.29	0.29	2.24	<0.05
C	1,190	21,394	25,207	54.1	0	NS
D	5,171	6.28	6.78	7.85	0.01	NS
E	5,712	2.35	59,928	0.2	8.5	<0.001
F	4,381	3.64	593.04	0.5	4.98	<0.01
G	4,621	2.31	34.46	0.64	3.78	<0.01
H	1,952	3.27	91.21	0.68	1.61	NS
I	5,744	1.94	22,952	0.17	4.21	<0.01
K	6,283	2.24	26.39	0.65	3.17	<0.05
L	5,319	2.04	>2.9E10	0.05	9.78	<0.001
M	2,723	2.03	570.3	0.28	2.51	<0.05
N	5,085	31.76	22.73	0.39	2.15	<0.05
P	4,073	2.12	66.77	0.46	3.04	<0.05
Q	3,677	1.99	4,928	0.23	5.71	<0.001
R	2,299	2.45	12.47	1.03	0.75	NS
S	7,480	1.75	7.01	0.82	0.88	NS
T	4,949	1.65	3,353	0.18	5	<0.01
V	4,860	1.31	1,501	0.15	4.1	<0.01
W	1,706	2.83	>4.8E10	0.05	4.25	<0.01
Y	3,035	2.96	10.65	1.52	0.83	NS

^a^Outgroup conserved sites only.

In contrast, analysis of sites that are conserved between *S. cariocanus* and *S. cerevisiae* showed a very different spectrum of deleterious effects with substantial variation among sites (e.g., 36% of sites are predicted as strongly deleterious and only 15% effectively neutral; fig. 3), motivating further partitioning of effects in *S. paradoxus*, and again, no advantageous effects. We have tested the significance of selective constraint on the distribution of deleterious fitness effects using likelihood and found it highly significant (models where outgroup conserved and nonconserved sites are considered separately vs. models with the two types pooled; from table 1, Model 1: 2ΔLogLikelihood = 2[−2,586.32 − (−2,666.78)] =160.92, df = (2*1) − 1 = 1, *P* < 10^−^^5^ or Model 2: 2ΔLogLikelihood = 2[−2,517.14 − (−2,603.3)] = 172.32, df =(2*2) − 2 = 2, *P* < 10^−^^5^, for separate vs. pooled effects, respectively).

### The Effect of Mutations on Individual Nucleotides and Amino Acids

**Fig. 4.—:**
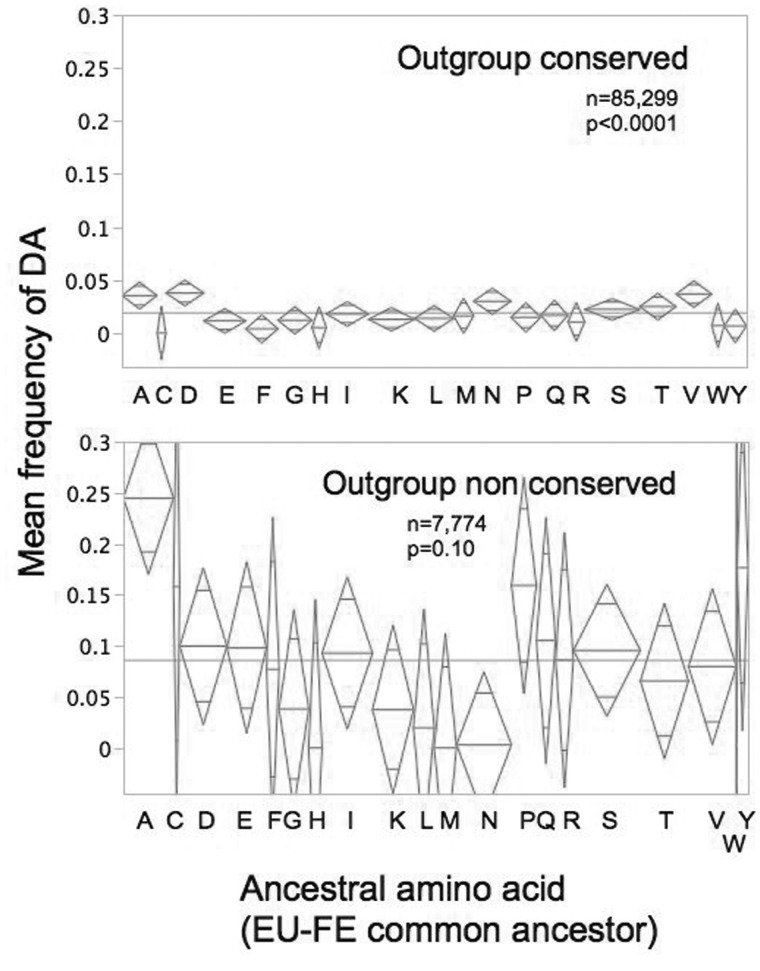
Effects of ancestral amino acid on frequency of DAs (diamonds are centered on sample means, with upper and lower edges showing the 95% confidence intervals; diamond width is proportional to sample size; horizontal line marks sample means). Wilcoxon, nonparametric tests for differences in frequencies of DAs between different amino acids in the EU-FE ancestral protein: Outgroup conserved sites: *P* < 0.0001; Outgroup nonconserved sites: *P* = 0.06.

We next asked whether the frequency of DAs at a site differs according to whether the ancestral sequence had a G or C or A or T, or by the translated amino acid at that site. For ancestral nucleotides, there is a significant interaction of effects, such that DA frequency is influenced by type of ancestral nucleotide, but only at nondegenerate or partly degenerate sites, there being no effect for the 4-fold degenerate sites (chi square: *P* < 0.001 for 0- and 2-fold degenerate sites vs. *P* = 0.07 for 4-fold degenerate), implying the effect of ancestral nucleotide is due to nucleotides affecting amino acid-changing codons, with little residual effect for silent nucleotides, that is, selection is on the amino acid, not the nucleotide. Indeed, there is a strong effect of ancestral amino acid identity on the degree of tolerance for new mutations at different sites, particularly those at outgroup conserved sites (chi-square: *P* < 0.0001 and *P* = 0.08 for conserved and nonconserved sites, respectively; fig. 4). Sites at which the ancestor had aspartic acid (D), valine (V), alanine (A), or asparagine (N) have the highest frequencies of DAs, whereas those with cysteine (C) have the lowest.

These differences also appear in the estimates of fitness effects. A very high proportion of strongly deleterious effects is predicted for mutations at cysteine (C), tryptophan (W), leucine (L), and glutamic acid (E) sites (table 2 and fig. 3), whereas those at asparagine (N), lysine (K), arginine (R), tyrosine (Y), serine (S), and aspartic acid (D) appear substantially milder. Still, for most amino acids, the model including a gamma distribution of effects is significant, indicating a wide range of effects upon mutating even single amino acids. Again, likelihood tests indicate a highly significant effect of amino acid identity on the distribution of deleterious fitness effects (models where each amino acid is considered separately vs. models with all amino acids combined; Model 1: 2ΔLogLikelihood = 2 [−1,956.07 − (−1,995.85)] = 79.56, df = (20*1) − 1 = 19, *P* < 10^−^^5^ or Model 2: 2ΔLogLikelihood = 2 [−1,884.57 − (−1,926.67)] = 82.86, df = (20*2) − 2 = 38, *P* = 3.5 × 10^−^^5^, for separate vs. pooled effects, respectively). Chemical properties of amino acid molecules such as size, polarity, hydrophobicity, and charge presumably are interacting with protein secondary structure and other structural considerations in the protein molecule to determine the sign and magnitude of effects. Previous studies have suggested that β sheets are the least tolerant to mutational changes (Nilsson et al. 2011; Melamed et al. 2014).

### Using the Estimated Short-Term Effects of New Mutations to Predict Long-Term Sequence Divergence

**Fig. 5.—:**
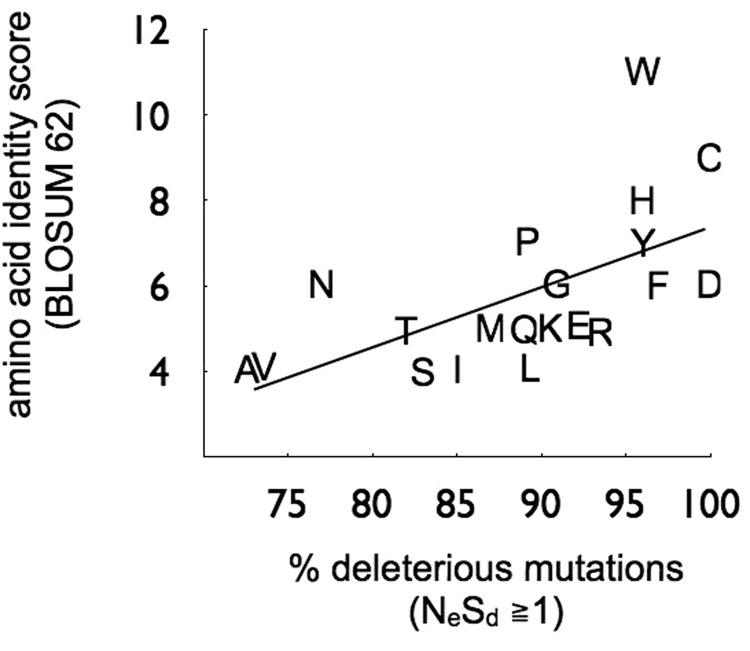
Long-term stasis of amino acids as a function of short-term tolerance to mutations. Amino acid identity scores computed from protein alignment blocks with greater than 62% identity (BLOSUM 62 matrix; Henikoff S and Henikoff JG 1992) plotted against proportion of deleterious effects of mutations changing that amino acid (NeSd ≥ 1; outgroup conserved sites only; df = 19, *r*^2 ^= 0.35, *P* = 0.006).

Long-term sequence divergence is thought to be the product of the cumulative effects of short-term processes occurring in populations, with sites that are least tolerant to new mutations being less “evolvable” and thus more likely to show evolutionary stasis. We may therefore expect to be able to predict the extent of conservation among sites occupied by particular amino acids from the magnitude of deleterious effects of new mutations at those sites. To test this expectation, we have correlated for each ancestral amino acid, the estimated proportion of deleterious effects upon mutating that amino acid in the European population of *S. paradoxus*, with a measure of the amino acid’s conservation during long-term overall divergence among sequences from a variety of organisms. For this we have used the BLOSUM 62 amino acid identity score, a measure of the probability of an amino acid remaining unchanged in an alignment block of diverse sequences with at least 62% identities (Henikoff S and Henikoff JG 1992). We find a significantly positive correlation between the two measures (outgroup conserved sites only; *r*^2 ^= 0.35; *P* = 0.006; fig. 5), indicating that long-term amino acid stasis can indeed be predicted by the magnitude of short-term purifying selection on nucleotide sites. Somewhat analogous correlations have been reported between mutational intolerance of sites within a yeast protein domain tested in one species, assessed in vitro, and long-term evolutionary conservation (Koufopanou and Burt 2005; Melamed et al. 2014).

## Conclusions

Changes in DNA sequences can have a variety of effects, from strongly advantageous, to neutral, through to slightly deleterious or lethal, and it is a reasonable and worthwhile scientific goal to be able to predict some of these effects. The analytical method of Schneider et al. (2011) represents a major advance in the development of a predictive theory estimating the fitness effects of new mutations, as it allows us to measure fitness effects of new mutations from population genomic data, and therefore in the organisms’ natural environments. The method allows estimation of both beneficial and deleterious effects. Excepting a few studies in *Drosophila* and humans, reviewed below, we are not aware of another study attempting to estimate and partition fitness effects of new mutations among different categories of sites in a natural population.

As for most population genetic theory, the model of Schneider et al. was developed with an obligate sexual organism in mind, that is, *Drosophila* or humans, and is therefore not directly applicable to organisms with mixed life cycles such as yeast (Tsai et al. 2008). Simulation studies suggest that our main parameter estimates are robust to mild levels of linkage and population subdivision, but further studies modeling fitness effects in organisms with mixed life cycles would be desirable. Further analytical work allowing estimation of confidence limits on the various parameter estimates would also be useful. Finally, our study has used the 4-fold degenerate sites as a neutral standard for comparisons, even though there is some suggestion that these are less divergent and polymorphic than LTRs, and thus not entirely neutral. Actual beneficial effects are likely to be smaller than our estimates, and deleterious effects larger.

Consistent with previous studies (Liti et al. 2009; Elyashiv et al. 2010; Gossmann et al. 2012), we found no evidence for beneficial mutations in yeast. The best candidates for sites with beneficial effects would be among those sites that have changed in other closely related taxa. Even at outgroup nonconserved sites, however, there is no evidence for beneficial mutations, though we cannot rule out some low frequency of adaptive mutations. The lack of evidence for beneficial mutations in yeast contrasts with results from *Drosophila* and humans (Eyre-Walker and Keightley 2007; Gossmann et al. 2012). Perhaps an overall lack of environmental challenge due to lack of infectious, coevolving parasites, combined with isogamy and low rates of outcrossing contribute to a slow rate of evolution with little adaptive change. It is genes involved in immunity and sexual selection that often show evidence of adaptive change in other species (Obbard et al. 2009; Vacquier and Swanson 2011).

For deleterious mutations, the method allows us to test the null hypothesis of equal effects against the alternative of gamma-distributed effects. Not surprisingly, the model of equal effects does not provide a good fit to the complete data set, suggesting that there is “unexplained” variation. The fit gets better as the data are subdivided into smaller and smaller groups, indicating significant differences between groups. We found significant differences between sites that are outgroup conserved versus nonconserved. Mutations at nonconserved sites are predicted to be effectively neutral (estimated frequency of deleterious mutations with N_e_S_d_ ≥ 1 is zero), with no significant variation among sites, whereas mutations at outgroup conserved sites cause variable degrees of harm. Partitioning the sites into groups according to ancestral amino acid identity explains some of the variation, with mutations at certain amino acids being unconditionally deleterious, others unconditionally nearly neutral, and others with substantially variable effects.

We further showed that the proportion of mutations that are deleterious for an amino acid is positively correlated with the degree of conservation of that amino acid, as indicated in a BLOSUM matrix. As these matrices are derived using sequences from many taxa, this correlation indicates that the differences in mutational effects among amino acids are likely not restricted to *Saccharomyces*. Furthermore, the fact that the predicted proportions correlate well with another independent set of evolutionary rate differences across diverse life forms increases our confidence in the analysis. In taxa where there is an appreciable number of beneficial mutations, it will be interesting to see whether amino acid identity is also correlated with the rate of change. Ultimately, we would like to be able to develop a predictive theory to account for variation in the fitness effects of new mutations: An achievable, if long-term goal.
